# Recent insights into breast milk microRNA: their role as functional regulators

**DOI:** 10.3389/fnut.2024.1366435

**Published:** 2024-04-16

**Authors:** Yi-Ran Xu, Jinglu Zhao, Hsi-Yuan Huang, Yang-Chi-Dung Lin, Tzong-Yi Lee, Hsien-Da Huang, Yi Yang, Yong-Fei Wang

**Affiliations:** ^1^Warshel Institute for Computational Biology, The Chinese University of Hong Kong, Shenzhen, Guangdong, China; ^2^School of Medicine, The Chinese University of Hong Kong, Shenzhen, Guangdong, China; ^3^Institute of Bioinformatics and Systems Biology and Center for Intelligent Drug Systems and Smart Bio-devices (IDS2B), National Yang Ming Chiao Tung University, Hsinchu, Taiwan; ^4^Department of Nephrology, Center for Regeneration and Aging Medicine, The Fourth Affiliated Hospital of School of Medicine, and International School of Medicine, International Institutes of Medicine, Zhejiang University, Yiwu, China; ^5^Zhejiang-Denmark Joint Laboratory of Regeneration and Aging Medicine, Yiwu, China

**Keywords:** miRNA, breast milk, infant development, immune system, digestive system

## Abstract

Breast milk (BM) is a primary biofluid that plays a crucial role in infant development and the regulation of the immune system. As a class of rich biomolecules in BM, microRNAs (miRNAs) are regarded as active factors contributing to infant growth and development. Surprisingly, these molecules exhibit resilience in harsh conditions, providing an opportunity for infants to absorb them. In addition, many studies have shown that miRNAs in breast milk, when absorbed into the gastrointestinal system, can act as a class of functional regulators to effectively regulate gene expression. Understanding the absorption pattern of BM miRNA may facilitate the creation of formula with a more optimal miRNA balance and pave the way for novel drug delivery techniques. In this review, we initially present evidence of BM miRNA absorption. Subsequently, we compile studies that integrate both *in vivo* and *in vitro* findings to illustrate the bioavailability and biodistribution of BM miRNAs post-absorption. In addition, we evaluate the strengths and weaknesses of previous studies and discuss potential variables contributing to discrepancies in their outcomes. This literature review indicates that miRNAs can be absorbed and act as regulatory agents.

## 1 Introduction

Breast milk (BM) is basically an essential source of nutrients that nourish and support the growth and development of infants. BM can be categorized into cellular, fat, and skim components (1). The number of milk-derived microRNAs (miRNAs) varies significantly among different components and across different species (Table 1). However, some miRNAs are shared in a common (2–4). Various factors could influence miRNA profiles in BM (3), such as lactation periods (4), gestation age (5), sex of infant (6), maternal weight (7), and diet, particularly high-fat diets (8). It's worth noting that milk-derived miRNA profiles undergo significant changes during lactation periods (4). These characteristics remain consistent across a range of species, including human (5), bovine (9), porcine (10), tammar wallaby (11), and more (Table 1). This consistency implies the potential for biological function.

**Table 1:** A summary of types of miRNAs detected in milk composition across species.

**Species**	**Milk fraction**	**Types of miRNAs detected**	**Profiling method**	**References**
Human	Skim milk (mature)	429	RT-qPCR	(2)
	Skim milk (colostrum)	386	RT-qPCR	(2)
	Cellular + lipid fraction	681	Taqman OpenArray Panel system	(12)
	Skim milk (mature)	281	Microarray	(6)
	Milk lipids	308	Small RNA sequencing	(3)
	Milk cell pre-feed	1,287	Small RNA sequencing	(4)
	Milk cell post-feed	1,308	Small RNA sequencing	(4)
	Milk exosomes (mature)	602	RT-qPCR	(13)
	Milk exosomes	610	Small RNA sequencing	(14)
Bovine	Skim milk (colostrum)	230	Small RNA sequencing	(15)
	Skim milk (mature)	213	Small RNA sequencing	(15)
	Skim milk (colostrum)	100	Microarray	(16)
	Skim milk (mature)	53	Microarray	(16)
	Fat + skim	363	RT-qPCR	(17)
	Milk EVs	276 + 503 (novel)	Small RNA sequencing	(18)
Porcine	Milk exosomes (colostrum)	491	Small RNA sequencing	(18)
	Milk exosomes	234	RT-qPCR	(10)
Murine	Skim milk (colostrum)	128	Microarray	(19)
	Skim milk (mature)	144	Microarray	(19)
Sheep	Milk EVs	84 + 601 (novel)	RT-qPCR	(20)
Giant panda	Milk exosomes	1,191	RT-qPCR	(21)

It has been shown that milk-derived miRNAs can survive in harsh conditions, including low pH (19, 22) environments (13, 23), such as the freezing-thawing cycle (22, 24) and digestive system. This resilience could be attributed to the protective role of milk exosomes (25), suggesting a potential way for drug delivery. Besides, before internalization, miRNA must cross several physical barriers, including the gastrointestinal (GI) tract and its mucus layer (26). Regarding the protective mechanisms, several studies have proposed different theories. As summarized by Carrillo-Lozano et al., extracellular vesicles, such as exosomes, protect miRNAs from harsh conditions in skim and lipid fractions (27). Other possible molecules, such as fat globules, Argonaute-2, RICS-Complex, and even mammary epithelial cells, may also be involved in BM miRNA protection (23).

**Figure 1:**
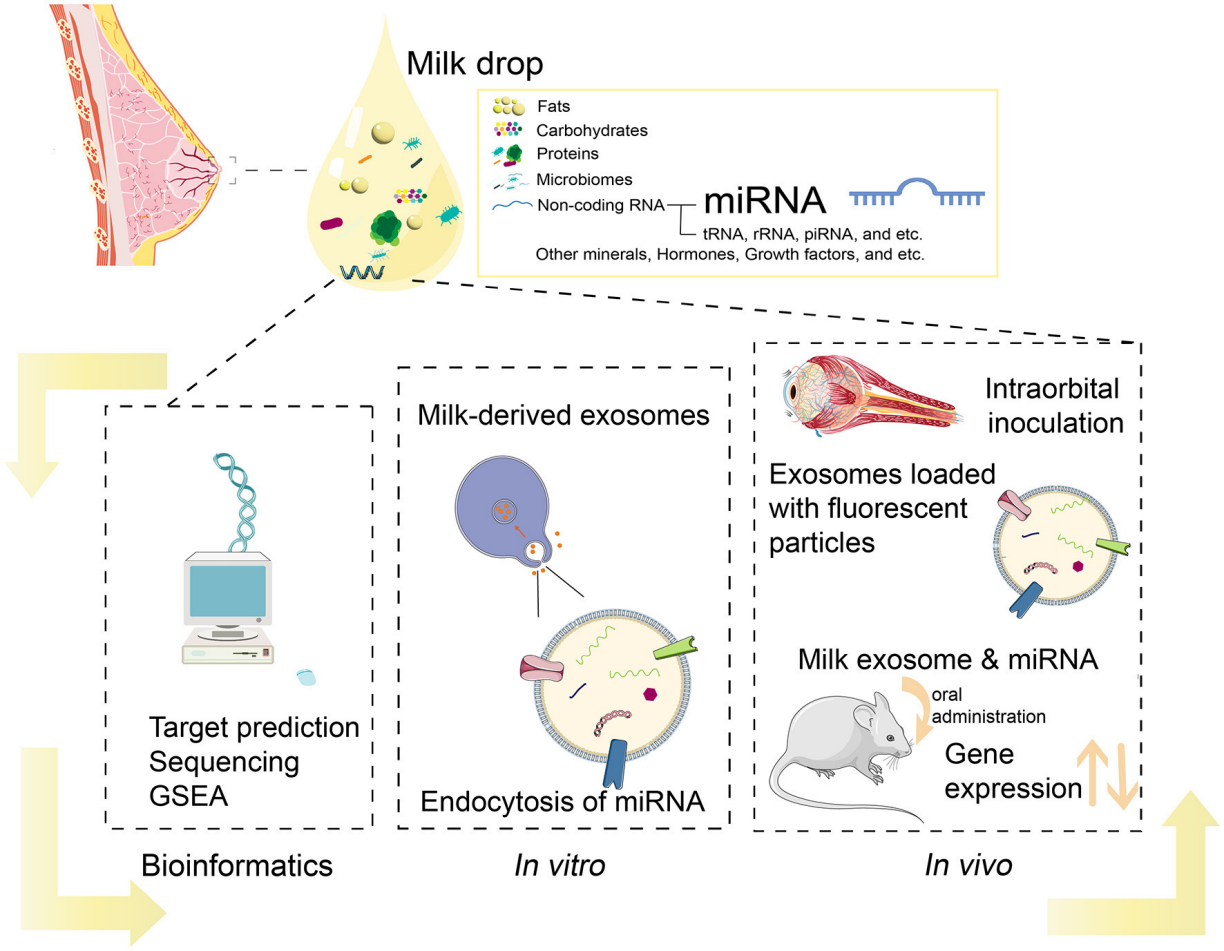
Current methods in analysis of BM miRNAs.

Recent studies indicate that BM miRNAs could act as functional regulators (Figure 1), impacting infant immunity and development (5, 21, 24). For example, *miR-223* is a regulator of granulosa lineage cell commitment, and its expression level is not affected by pasteurization, but it is highly expressed in both colostrum and mature human milk (28, 29). Besides, both *miR-let-7a-5p* and *miR-181-5p* were found to be highly enriched in BM across species. *MiR-let-7a-5p* is an important regulator of inflammatory response and cellular phenotype (30), whereas *miR-148a-3p* plays an essential role in controlling inflammation and affecting cancer development (31). In addition, previous studies have suggested that miRNAs in BM could be involved in infant development, including the regulation of intestinal function (4) and neurogenesis (32–35). Moreover, miRNAs in BM have also been associated with lipid metabolism (36, 37). Taken together, these studies suggest a crucial role of BM miRNAs in infants' immune systems and development.

However, questions persist regarding the mechanism of BM miRNA absorption, and their bioavailability within the infant's internal environment. So far, two hypotheses exist to explain the function of miRNA in BM: the functional hypothesis, which posits that miRNAs can retain biological activity after absorption; but the nutritional hypothesis, which suggests that miRNAs are degraded before absorption and primarily serve as nutritional components (38).

Several reviews have endeavored to summarize and discuss recent research on the uptake of dietary miRNAs. Some of them focused on bioinformatics analysis on BM miRNA profiles and phenotypic changes after milk meal, revealing many potential benefits of BM on infants, even across species. In this review, we predominantly emphasized experimental studies validated by both *in vivo* and *in vitro* studies (Supplementary material). These findings demonstrate the absorption of BM miRNAs, along with their bioavailability and distribution following uptake, showing that BM miRNAs are transferable genetic materials.

## 2 Absorption of BM-derived miRNAs

Previous studies suggest that the BM miRNAs have the potential to be absorbed by varied cell lines. Wolf et al. employed a fluorescence-based approach to detect the *in vitro* absorption of bovine milk in both Caco-2 intestinal epithelial cells (IEC) and IEC-6 cells. The uptake of BM exosomal miRNAs could be mediated by endocytosis and appeared to be affected by both cell and exosome surface protein (39, 40). This is indicated by a significant decrease in uptake efficacy when the proteins on the surface of BM exosomes are removed or when Caco-2 cells are treated with proteinase K (39). Wolf et al. also demonstrated that exosomes underwent unidirectional transport across intestinal monolayers, moving from the apical chamber to the basolateral chamber, with minimal reverse transportation (39). The uptake was also found to be dependent on temperature, with more than a 50% decrease in uptake observed when the temperature drops from 37 to 4°C (39).

However, it appears that environmental pH levels may have a limited impact on the absorption of BM-derived miRNAs. Liao et al. conducted an experiment in which fluorescence-labeled human milk exosomes were incubated with human intestinal crypt-like cells (HIEC) (22). They observed an increase in fluorescence density within HIEC after a 2-h incubation at pH 4. However, there was no statistically significant difference in the change of fluorescence density at pH 2, suggesting that variations in environmental pH may not significantly influence the uptake of these miRNAs.

It's worth noting that 10% of the internalized human milk exosomes were identified within the nucleus of HIEC, providing evidence of the potential impact of human milk miRNA on cellular regulation (22). This internalization and nuclear localization can also be seen from confocal microscopy (41). More importantly, both *miR-21-5p* and *miR-30a-5p* derived from bovine milk were identified in human plasma following a meal, and these detectable levels persisted for up to 6 h (42). Taken together, these studies have provided compelling evidence that BM miRNA derived from one species can be detected within the cells of another species, strongly supporting the functional hypothesis.

Although the absorption of BM exosomal miRNA is well-studied, it has been reported that non-exosomal BM miRNAs could also be taken up in the digestive system (43). Generally speaking, milk can be separated into different components, including milk fat, whey, casein, cells, and debris, through the process of differential centrifugation. Through employing ultra-centrifugation, it is possible to achieve more refined separation, enabling the isolation of extracellular vesicles (EVs) from the supernatant (44). Lin et al. demonstrated that certain milk-derived miRNAs, such as *miR-2291* and *miR-7134*, displayed unique expression patterns in exosomes compared to the exosome-free supernatant where no such miRNAs are found (43). By examining these exosomal and non-exosomal specific miRNAs in IPEC-J2 cells following incubation with milk exosomes and exosome-free supernatants, their study revealed that both exosomal and non-exosomal miRNAs could be absorbed by the IPEC-J2 cells.

Extracellular vehicles are essential for the uptake of BM miRNAs. They not only deliver miRNAs to target cells, but also significantly enhance miRNAs stability under harsh conditions. Chen et al. suggested that SID-1 transmembrane family member 1 (SIDT1) might act as a transporter for BM miRNA uptake (45). Furthermore, Wei et al. showed that endocytosis of exosomal miRNAs is mediated by caveolae- and lipid raft-dependent pathways (46). Although various mechanisms and sites of uptake have been proposed, such as intestinal epithelial cells and vascular epithelial cells (39, 40), it's widely accepted that BM miRNAs can be absorbed and function as regulators.

## 3 Bioavailability of BM-derived miRNA

Considering the uptake of BM miRNA, questions have emerged regarding whether miRNAs absorbed from BM can reach their target sites and directly impact gene expression. So far, several studies have given positive answers to the questions by cooperating *in vitro* and *in vivo* experiments. The core methodology of these studies is consistent: they track changes in specific miRNA attributes or biomarkers *in vivo* and then explore the resulting shifts in cellular metabolism caused by these altered miRNA traits in a controlled *in vitro* setting. Therefore, in conducting *in vitro* experiments, it's crucial to carefully consider confounding factors such as the duration miRNAs remain stable in the *in vivo* environment before degrading, and the real concentration of miRNAs that are absorbed into the system.

Chen et al. not only showed that porcine milk-derived miRNAs, including *miR-7134, miR-1343, miR-2320, miR-181a, miR-769-3p*, and *miR-128*, were absorbed by porcine intestinal cell line (IPEC-J2), but also observed a down-regulation of *FAS* and *SERPINE* in the cell line after the uptake of miRNAs (47). These genes are targets of the aforementioned miRNAs.

Similarly, Baier et al. showed a significant absorption of *miR-29b* and *miR-200c* through the administration of varying milk doses to individuals in a randomized crossover design study. In addition, they also observed significant changes in the expression levels of miRNAs' target genes (48). To investigate the bioactivity of the absorbed miRNAs further, they performed a luciferase reporter gene assay. These reporter genes incorporated 3′UTR regions containing specific binding sites for these miRNAs (48). The assay revealed a substantial decrease in the activity of reporter genes when HEK-293 cells were cultured with milk exosomes containing *miR-29b* and *miR-200c* that mimicked postprandial concentrations, suggesting the function of milk-derived miRNAs after absorption (48). The detection of *miR-1* is also involved in this study, as it was not detectable in milk and served as a negative control, which is one of the key points for the experiment. Another notable aspect is that they added the milk exosomes to the cell culture media mimicking the postprandial concentrations of *miR-29b* and *miR-200c* (48). However, Auerbach et al. failed to replicate these results and pointed out that this contradiction might be attributed to technique variations, such as the differences in RNA purification, qPCR assay design and other factors (49).

In another study, researchers revealed the influence of BM *miR-26a* on the development of offspring's adipose tissue (50). Their findings demonstrated that changes in the level of *miR-26a*, delivered through milk, can alter the expression of target genes in the offspring, subsequently affecting adipose tissue development (50). The key aspect of this discovery relied on the selection of *miR-26a*, which is among the 10 most abundant miRNAs in breast milk, as indicated by several studies (8, 22, 41, 51, 52). Nevertheless, it is also essential for future studies to consider the *in vivo* metabolism and circulation of BM *miR-26a*.

Bioinformatics analysis also offered indirect support for the functional hypothesis. For example, upregulated miRNAs in moderate/very preterm compared to term mature milk tended to be enriched in the neuro-related GO pathways (53). This result may indicate that BM miRNAs play a crucial role in infants' neurodevelopment (54). In addition, the target genes of the most abundant miRNAs in BM were found to be enriched in immune-related pathways, such as TGF-beta signaling, T-cell receptor signaling, Toll-like receptor signaling, Jak-STAT signaling, and Th1 and Th2 cell differentiation (55). Other studies employing comparable methodologies have supplied evidence suggesting that miRNAs may function as regulators in various domains, including neurogenesis, gut maturation, epigenetics, and infant metabolism and development (56). Collectively, these studies suggest the functional hypothesis of BM miRNAs.

## 4 Biodistribution of milk-derived miRNA

Current studies show that the milk exosomes were predominantly concentrated in the liver and spleen following uptake (40, 57). Kusuma et al. observed that the majority of exosomes were cleared from the circulation and distributed in a region near the liver within 18 h after intra-orbital injection of DiR-labeled milk exosomes (40). Manca et al. also reported that the liver and spleen were the primary organs enriched with milk-derived exosomes following a milk meal (57). Small extracellular vesicles were also estimated to accumulate in the intestinal mucosa, liver, brain, bone, and thymus, with a bioavailability of up to 45% after oral administration (58).

The distribution of exosomal miRNA exhibited variations may vary depending on different administration methods (such as intravenous injection or oral administration) and the types of miRNA (57). Munagala et al. (48) showed that the presence of milk exosomes could also be detected in other organs that were not reported by the previous studies, regardless of administration methods (59). This inconsistency could be attributed to the absence of controls (free DiR or unlabeled exosomes) and the notable difference in oral miRNA doses, which were four times higher compared to the previous study (57).

Although these studies point to the biodistribution of exosome uptake, their function in particular organs remains unknown. Considering that macrophages in the liver and spleen are responsible for clearing foreign exosomes administered to mice (57), it suggests a likelihood that milk-derived exosomal miRNAs could exert their effects before undergoing clearance by macrophages. Moreover, the failure of bovine milk exosomes to rescue Drosha knockout mice—genetically modified mice with a loss of microRNA maturation—indicates the need for further investigation to comprehend the biological efficacy and absorption levels of milk-derived miRNAs (57).

## 5 Discussion

In this review, we follow the logic of “absorption-bioavailability-biodistribution” to provide evidence supporting the functional hypothesis. The discussion of bioavailability and biodistribution is based on the fact that miRNAs in breast milk are indeed absorbed by infants, and aims to discuss how and where they have biological effects on gene expression. Based on these studies, we conclude that miRNAs can be absorbed and exert biological function.

However, some confounding factors should be carefully assessed. The selection of miRNA as evidence of absorption demands careful consideration due to its inherent properties. For example, internal miRNA profile could influence the results. The disparities in milk-derived miRNA biodistribution between mice after oral administration and intravenous injection suggest that oral milk meals may indeed induce a series of changes in miRNA profiles (57). Therefore, using labeled exogenous miRNAs that cannot be naturally produced by the host would be a preferable method for detecting the absorption of BM miRNA. This approach helps eliminate the potential confounding factors associated with endogenous miRNA changes and provides a more accurate assessment of the specific miRNAs introduced through the diet.

Transgenic mice are also used to validate the absorption of BM-derived miRNAs by examining changes in endogenous miRNA expression after *in vitro* incubation or *in vivo* uptake. However, this approach may include unknown variables that could affect the experiment results. For example, the miRNAs being uptaken may not exclusively originate from the milk meal and could also come from other gastric contents (60).

In addition, Wang et al. (42) suggested that using heparin tubes for blood collection can entirely eliminate the miRNAs in the sample, while the hemolysis of human red blood cells can significantly increase *miR-16* levels in the sample (42). This underscores the importance of employing the correct method for collecting blood samples when detecting miRNA after a milk meal.

Studies of the absorption and bioavailability of milk-derived miRNAs have significant importance. Understanding this process could facilitate the development of artificially modified milk compositions containing a more balanced array of miRNAs, thus potentially enhancing infant development. it provides a natural solution that surpasses the traditional use of artificial nanoparticles as carriers for RNA interference (RNAi) drugs (26, 57). Once it is confirmed that BM miRNAs are functional regulators, more comprehensive testing of commercial milk may need to be considered to ensure the quality of commercial milk at the molecular level (61). Therefore, several issues or questions should be considered in future studies: (1) Although the results from *in vivo* experiments consistently demonstrate the absorption of milk-derived miRNA from the digestive system, there is a disparity in the conclusion derived from *in vivo* experiments. More careful selection of miRNAs as biomarkers is needed. (2) Further investigation is required to ascertain the bioavailability and adequacy of milk-derived miRNAs in altering gene expression after absorption (57). To assess the bioavailability of milk-derived miRNAs, it is imperative to investigate the metabolism of these miRNAs after a milk meal. (3) Understanding the mechanism of miRNA absorption is essential, including whether the gastrointestinal tract possesses specific sites for the utilization of milk-derived miRNA and the potential role of miRNAs in the gastrointestinal tract (62). This is necessary as it may explain the lack of observed evidence regarding the absorption of milk-derived miRNA into organs or circulation. (4) Apart from cellular uptake in the digestive tract itself, it is also plausible that the microbiome could involve the process of milk-derived miRNA absorption.

## Author contributions

Y-RX: Conceptualization, Data curation, Formal analysis, Funding acquisition, Investigation, Methodology, Resources, Software, Validation, Visualization, Writing – original draft, Writing – review & editing. JZ: Investigation, Visualization, Writing – review & editing. H-YH: Writing – review & editing, Methodology. Y-C-DL: Writing – review & editing, Methodology. T-YL: Writing – review & editing, Methodology. H-DH: Writing – review & editing, Methodology. YY: Writing – review & editing, Funding acquisition, Resources, Supervision. Y-FW: Conceptualization, Funding acquisition, Project administration, Resources, Supervision, Validation, Writing – original draft, Writing – review & editing.
