# Supplementary material for: Recent insights into breast milk microRNA: their role as functional regulators
Source: Front Nutr. 2024 Apr 16;11:1366435. doi: 10.3389/fnut.2024.1366435 (PMC11058965; doi:10.3389/fnut.2024.1366435)
Supplement: Supplementary file 1 [file Data_Sheet_1.PDF]

Supplementary Materials for Literature Search

Search Strategy

To ensure a comprehensive review of the literature related to this study, the strategy was designed to capture all relevant studies by using a combination of keywords, and the search was conducted based on the PubMed database.

Keywords and Search Terms

The following keywords and search terms were used in various combinations:

"milk", "miRNA", "exosome", "uptake", "absorption", and "profile". The keywords were combined by the Boolean operator AND & OR for comprehensive and specific searches. Several other papers were selected from the reference of these papers and "Similar articles".

Inclusion and Exclusion Criteria

Studies published from 2014-2023 were included. Only experimental studies were included. Review articles and Studies merely focusing on bioinformatics analysis on milk miRNA are excluded. The research focused only on the metabolism of exosomes without studying the miRNAs inside, which were also included as milk exosomes are one of the major transporters for miRNA. We filtered out those articles on the effect of dietary miRNAs but not specific to the milk diet. Studies that only provide phenotypic change after milk meal without providing detailed mechanisms or direct evidence of miRNA as transferable genetic materials are excluded as well.

Summary

Finally, 16 papers were left. Of those, three showed evidence that milk miRNAs cannot be uptake as functional regulators, while the other 13 studies showed the regulatory effects of milk miRNAs after absorption. Only 16 studies were left, as the majority of miRNA/exosome uptake research does not relate to milk components, and many provide predictions based on phenotypic change after milk meal but do not give direct evidence of BM miRNA uptake. The selected papers are shown in **Supplementary Table S1** below. Studies that provide evidence on either the Functional Hypothesis or the Nutrient Hypothesis are indicated in the last column.

Supplementary Table S1 Comparative Summary of Studies on the Functional vs. Nutrient Hypotheses

	Papers	Key conclusions/results in the studies	Viewpoint

**1**	Wolf et al., J Nutr., 2015	"This research assessed the transport mechanism of bovine milk exosomes, and therefore microRNAs, in human and rodent intestinal cells."(1)	Functional Hypothesis
**2**	Kusuma et al., Cell Physiol., 2016	"Test the hypothesis that human vascular endothelial cells transport milk exosomes by endocytosis." (2)	Functional Hypothesis
**3**	Kahn et al., Mol Nutr Food Res, 2018	"For the first time reveal the survivability of preterm milk exosomes following simulated gastric/pancreatic digestion" (3)	Functional Hypothesis
**4**	Liao et al., Mol Nutr Food Res., 2017	"Reveal the survivability and complexity of human milk exosome microRNAs upon simulated gastric/pancreatic digestion, and the dynamics during lactation stages."(4)	Functional Hypothesis
**5**	Wang et al., J Nutr., 2018	"Assessed and identified potential confounders of plasma miR analysis with the intent to develop a consensus of minimal experimental requirements in future studies of dietary miRs."(5)	Functional Hypothesis
**6**	Lin et al., Sci Rep., 2020	"Suggested that the milk miRNAs can be absorbed both in vivo and in vitro"(6)	Functional Hypothesis
**7**	Chen et al., Cell Res., 2021	"Reveal a major mechanism underlying the absorption of dietary microRNAs, uncover an unexpected role of the stomach and shed light on developing small RNA therapeutics by oral delivery."(7)	Functional Hypothesis
**8**	Chen et al., Sci Rep., 2016	"Demonstrated that milk-derived exosomes can facilitate intestinal cell proliferation and intestinal tract development"(8)	Functional Hypothesis
**9**	Baier et al., J Nutr., 2014	"MiRNAs in milk are bioactive food compounds that regulate human genes."(9)	Functional Hypothesis
**10**	Pomar et al., FASEB J., 2021	"Milk miR-26a may act as an epigenetic regulator influencing early metabolic program in the progeny, which emerges as a relevant component of an optimal milk composition for correct development"(10)	Functional Hypothesis

**11**	Manca et al., Sci Rep., 2018	“Assessed the bioavailability and distribution of exosomes and their microRNA cargos from bovine, porcine and murine milk within and across species boundaries.”(11)	Functional Hypothesis
**12**	Khanam et al., Int J Pharm., 2023	"Conclude that the apparent bioavailability of sMEVs is 45%, and sMEVs are transported to peripheral tissues in C57BL/6J mice” (12)	Functional Hypothesis
**13**	Munagala et al., Cancer Lett., 2016	"The first report to identify a biocompatible and cost-effective means of exosomes to enhance oral bioavailability, improve efficacy and safety of drugs.”(13)	Functional Hypothesis
**14**	*Title et al., J Biol Chem., 2015	"Nutritionally derived microRNAs are unlikely to cross the intestinal barrier and influence gene expression”(14)	Nutrient Hypothesis
**15**	*Laubier et al., RNA Biol., 2015	“Did not detect an increase in miR-30b in tissues of pups fed by transgenic females compared to pups fed by wild-type females.”(15)	Nutrient Hypothesis
**16**	*Auerbach et al., F1000Research., 2016	Dietary xenomiRs cannot be transfer of into the circulation of adult humans. (16)	Nutrient Hypothesis

References

1. Wolf T, Baier SR, Zempleni J. The Intestinal Transport of Bovine Milk Exosomes Is Mediated by Endocytosis in Human Colon Carcinoma Caco-2 Cells and Rat Small Intestinal IEC-6 Cells. J Nutr. 2015 Oct;145(10):2201–6.

2. Kusuma RJ, Manca S, Friemel T, Sukreet S, Nguyen C, Zempleni J. Human vascular endothelial cells transport foreign exosomes from cow's milk by endocytosis. Am J Physiol Cell Physiol. 2016 May 15;310(10):C800–7.

3. Kahn S, Liao Y, Du X, Xu W, Li J, Lönnerdal B. Exosomal MicroRNAs in Milk from Mothers Delivering Preterm Infants Survive in Vitro Digestion and Are Taken Up by Human Intestinal Cells. Mol Nutr Food Res. 2018 Jun;62(11):e1701050.

4. Liao Y, Du X, Li J, Lönnerdal B. Human milk exosomes and their microRNAs survive digestion in vitro and are taken up by human intestinal cells. Molecular Nutrition & Food Research. 2017;61(11):1700082.

5. Wang L, Sadri M, Giraud D, Zempleni J. RNase H2-Dependent Polymerase Chain Reaction and Elimination of Confounders in Sample Collection, Storage, and Analysis Strengthen Evidence That microRNAs in Bovine Milk Are Bioavailable in Humans. J Nutr. 2018 Jan 1;148(1):153–9.

6. Lin D, Chen T, Xie M, Li M, Zeng B, Sun R, et al. Oral Administration of Bovine and Porcine Milk Exosome Alter miRNAs Profiles in Piglet Serum. Sci Rep. 2020 Apr 24;10:6983.

7. Chen Q, Zhang F, Dong L, Wu H, Xu J, Li H, et al. SIDT1-dependent absorption in the stomach mediates host uptake of dietary and orally administered microRNAs. Cell Res. 2021 Mar;31(3):247–58.

8. Chen T, Xie MY, Sun JJ, Ye RS, Cheng X, Sun RP, et al. Porcine milk-derived exosomes promote proliferation of intestinal epithelial cells. Sci Rep. 2016 Sep 20;6:33862.

9. Baier SR, Nguyen C, Xie F, Wood JR, Zempleni J. MicroRNAs are absorbed in biologically meaningful amounts from nutritionally relevant doses of cow milk and affect gene expression in peripheral blood mononuclear cells, HEK-293 kidney cell cultures, and mouse livers. J Nutr. 2014 Oct;144(10):1495–500.

10. Pomar CA, Serra F, Palou A, Sánchez J. Lower miR-26a levels in breastmilk affect gene expression in adipose tissue of offspring. The FASEB Journal. 2021;35(10):e21924.

11. Manca S, Upadhyaya B, Mutai E, Desaulniers AT, Cederberg RA, White BR, et al. Milk exosomes are bioavailable and distinct microRNA cargos have unique tissue distribution patterns. Sci Rep. 2018 Jul 27;8(1):11321.

12. Khanam A, Ngu A, Zempleni J. Bioavailability of orally administered small extracellular vesicles from bovine milk in C57BL/6J mice. International Journal of Pharmaceutics. 2023 May 25;639:122974.

13. Munagala R, Aqil F, Jeyabalan J, Gupta RC. Bovine milk-derived exosomes for drug delivery. Cancer Lett. 2016 Feb 1;371(1):48–61.

14. Title AC, Denzler R, Stoffel M. Uptake and Function Studies of Maternal Milk-derived MicroRNAs. J Biol Chem. 2015 Sep 25;290(39):23680–91.

15. Laubier J, Castille J, Le Guillou S, Le Provost F. No effect of an elevated miR-30b level in mouse milk on its level in pup tissues. RNA Biol. 2015;12(1):26–9.

16. Auerbach A, Vyas G, Li A, Halushka M, Witwer K. Uptake of dietary milk miRNAs by adult humans: a validation study. F1000Res. 2016 Apr 22;5:721.
